# Prevalence of Mental Disorders in 6–16-Year-Old Students in Sichuan Province, China

**DOI:** 10.3390/ijerph120505090

**Published:** 2015-05-13

**Authors:** Yuan Qu, Hongyun Jiang, Ni Zhang, Dahai Wang, Lanting Guo

**Affiliations:** Department of Psychiatry, West China Hospital, Sichuan University, Chengdu, Sichuan 610041, China; E-Mails: quyuan20030966@163.com (Y.Q.); Jianghongyun2015@163.com (H.J.); zhangni20030966@163.com (N.Z.); dahai_youwen@163.com (W.D.)

**Keywords:** China, mental disorder, epidemiological, prevalence, children, adolescents, students

## Abstract

To investigate the point prevalence of mental disorders in school students, multistage cluster stratified random sampling and two-phase survey methods were used to identify 40 primary and middle schools. The students were screened using the Chinese version of the Child Behavior Checklist and diagnosed using the Mini International Neuropsychiatric Interview. The prevalence of behavioral problems was 19.13%. The prevalence of behavioral problems significantly differed by sex, age, city of residence, and caretaker. The six-month prevalence of any mental disorder was 15.24% (95% CI: 15.49%–16.97%). Psychiatric disorders were more prevalent in boys (17.33%) relative to girls (13.11%; *p* < 0.01). The prevalence of mental disorders significantly differed by community and caretaker, and 36.46% of students exhibited comorbidity. Results demonstrated important mental health issues, with a high incidence of comorbidities, in this population. Students’ mental health requires increased attention, particularly in poverty-stricken areas and left-behind children and adolescents.

## 1. Introduction

Mental disorders, including attention deficit disorder (ADHD), disruptive behavior disorder (DBD), mood disorders, tic disorder (TD), and anxiety spectrum disorder [1], strongly affect learning and quality of life in children and adolescents and are closely related to adult mental health [2,3]. Children’s self-cognition is limited, and parental assessment is subjective [4]; therefore, there are few applicable epidemiological methods available [5,6,7,8,9], and few epidemiological studies have examined mental disorders in children and adolescents, the results of which are inconsistent [10,11]. Previous studies conducted in Western countries have reported a prevalence of 7%–20% for psychiatric disorders in children and adolescents; this includes the USA (13.3%) [12], Denmark (11.8%) [13], the UK (15%) [14], New Zealand (20.7%) [15], Norway (7.0%) [16], Puerto Rico (19.8%) [17], and Italy (10.5%) [18]. In developing countries, this prevalence is estimated at 12.5%–22.5%, for example, Sub-Saharan Africa (19.8%) [19], Chile (22.5%) [20], Brazil (22.5%) [21], India (12.5%) [22], and Oman (13.9%) [23].

During the past five years, our data has shown 6.95%–23.1% detection rate for behavioral problems in children [24,25,26]. To our knowledge, Chinese epidemiological studies examining child mental health are limited to Hunan and Liaoning provinces. In 1990, the largest Chinese epidemiological study to examine mental disorders in children and adolescents since the country was founded reported a 14.9% prevalence rates. In 2005, another epidemiological study examining mental disorders in elementary and middle school students in Hunan revealed a 16.22% prevalence rate [27]. In 2007, a study conducted in Liaoning observed a 9.2% prevalence rate in students aged 6–16 years [28]. Another 2012 study included violent, juvenile male criminals in Hunan and Sichuan, of whom 82% and 61%, respectively, met the criteria for mental disorders [29]. Meanwhile, the left-behind children, defined as “children younger than 18 years of age who have been left behind at their original residence for at least 6 months while one or both parents migrate to other areas to work”, had produced severe mental health issues and social problems in China. There have been no large-scale epidemiological surveys of mental disorders in children and adolescents conducted in Sichuan or nationwide. This study was one of China’s first nationwide epidemiological projects, which aimed to examine mental disorders in children and adolescents in Sichuan. We conducted a two-phase epidemiological survey, performed questionnaire screening, and interviewed students aged 6–16 years. The Diagnostic and Statistical Manual of Mental Disorders, Fourth Edition (DSM-IV) diagnostic criteria were used to analyze the distribution of mental disorders in children, to determine the current situation regarding mental disorders in primary and middle school students, and provide scientific reference for the implementation of related health policies and prevention strategies.

## 2. Participants and Methods

### 2.1. Participants

Minimum sample size was calculated by the national prevalence of children with schizophrenia, based on data from the sixth national census (2010) of the overall population regarding proportions of children aged 6–16 years. There were 20,752 children and teenagers being enrolled in this study. Chengdu, Neijiang, Dazhou, and Bazhong, cities in Sichuan were selected as investigational sites for this study, according to economic status, as they represented different levels of economic development. In 2012, Gross domestic product (GDP) per capita was $10,373.89, $4,646.87, $3661.10, and $2030.12 in Chengdu, Neijiang, Dazhou, and Bazhong, respectively. Schools were chosen at random, according to the number of classrooms, primary schools, and middle schools in each city. Five urban and five rural schools were selected randomly from each region, with a total of 40 primary and middle schools included in the study. Between 1 and 3 classes from Grades 1–10 were randomly selected from each school, depending on the size of the school. Participants’ ages ranged from 6 to 16 years. According to the principles of fairness, voluntariness, and cooperation, only children and adolescents (and their caregivers) who had agreed to participate and signed the consent form were enrolled in the study. Valid retrieved data were available for 19,711 students, with a response rate of 95%. The study was conducted between June 31, 2013 and June 31, 2014.

### 2.2. Sociodemographic Characteristics

A general questionnaire concerning psychological factors in children, including name, sex, age, parental occupation, parental educational level, family type, economic status, family relationships, family medical history, physical and mental health status, family environment, and parental education methods, was compiled.

### 2.3. The Screening Tool 

The standardized Chinese version of the Child Behavior Checklist (CBCL; parental version) was used, with 113 questions selected from the behavioral problems section. The presence of one or more factors with scores higher than those of Chinese normative data in the Achenbach System of Empirically Based Assessment suggested behavioral problems. The CBCL was completed by parents or other caregivers; if no parents or guardians were available, the student completed the form.

### 2.4. Diagnostic Criteria and Tools 

Relevant DSM-IV diagnostic criteria and the Mini International Neuropsychiatric Interview for Children and Adolescents (MINI-KID), which is a short-set diagnostic interview questionnaire designed for the diagnosis of DSM-IV mental disorders, were used to confirm mental disorders [30]. The MINI-KID was developed by psychiatrists and clinicians in the United States and Europe and is widely applied in multi-center clinical trials and epidemiological studies to perform brief and accurate formalized psychiatric examinations. The questionnaire is suitable for children and adolescents aged 6–16 years and include versions for parents and children [31]. Previous studies have shown that the Chinese parental and children’s versions of the MINI-KID demonstrated high diagnostic sensitivity and is suitable for comprehensive psychiatric diagnosis in children and adolescents [32]. If a positive diagnosis is indicated in one version, the overall result is positive; however, a negative diagnosis is required from both versions for a negative result. This study used both versions, unless circumstances prevented this (e.g., if parents were unable to participate) or diagnosis was difficult. The timeframe for the MINI-KID was 6 months.

### 2.5. Study Procedures

**Figure 1:**
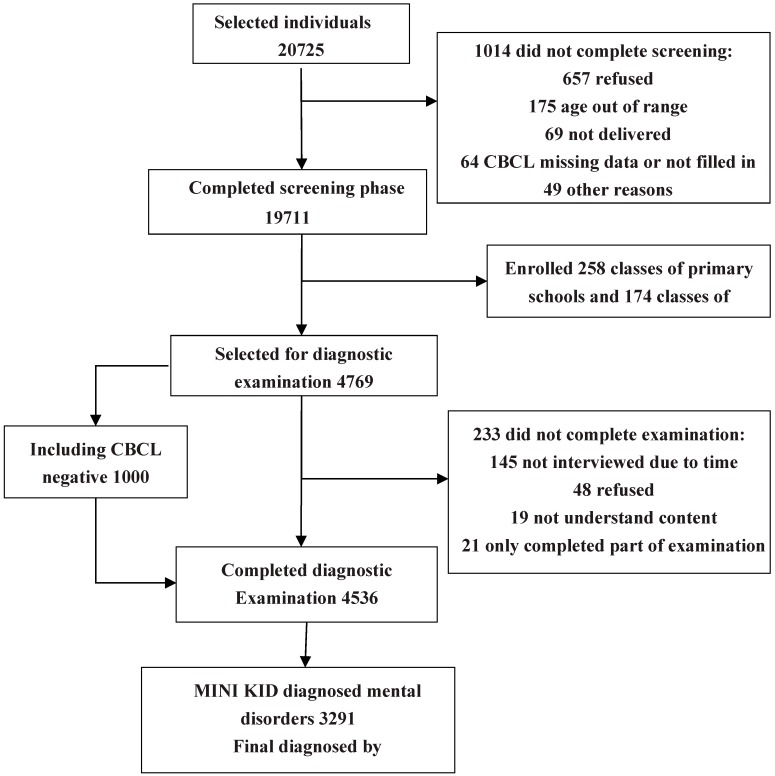
Subject flow chart.

The project was approved by the ethics review committee of the West China Hospital of Sichuan University. The first step included preparation of survey tools. The second step involved training and a pre-test. Investigators consisted of psychiatrists and graduate and undergraduate volunteers from medical institutions. Prior to the initiation of the study, investigators were trained to administer all questionnaires and procedures. Team leaders from Beijing trained the project’s researchers, who subsequently trained the volunteers. A consistency test was conducted to assess the diagnostic results obtained by the researchers, providing a kappa value of 0.85–0.91. During the pre-test stage, 800 students attending Grades 1–10 in one primary and one middle school were randomly selected to verify the validity and reliability of the CBCL, which has been found to be an effective instrument for assessing emotional and behavioral problems in children. Numerous studies have examined the CBCL in China, and results from our pre-test stage revealed good validity and reliability for use in the Chinese population. There was high consistency between MINI-KID results clinical diagnoses by medical professionals (χ^2^ = 52.16, *p* < 0.01), with a kappa value of 0.94. The third step involved an official large-scale investigation. Parents completed the CBCL questionnaire for screening, followed by a one-to-one standard formalized examination using the MINI-KID for parents of children with positive CBCL results. Clinical diagnosis was made by psychiatrists, based on DSM-IV criteria. Thereafter, 1000 cases with negative CBCL results were randomly selected from each class from the schools chosen to verify the diagnosis. The fourth step involved data organization and analysis. See Figure 1 for the sampling schematic diagram.

### 2.6. Statistical Analysis

Epidata 3.1 software was used to establish the database. All data were analyzed using SPSS 17.0 software. We estimated frequencies and 95% confidence intervals (CI) for individual mental disorders. Sample distributions of sex, age, region, caregiver, parental education level, family economic status, family structure, and family history of mental disease were calculated. The prevalences of mental disorders and comorbidity were calculated using frequency analysis with 95% CI. Subsamples were compared using t tests and chi-square tests. In subsequent analysis, individual disorders were clustered into 6 main diagnostic groups. Due to the relatively low prevalence of eating disorder, it was combined with all other mental disorders. Diagnoses related to ADHD, conduct disorder (CD), and oppositional defiant disorder (ODD) were categorized as disruptive disorders. A chi-square test was performed to compare age, sex, caregiver, and region, according to mental disorders, and examine differences in outcome. Statistical significance was based on two-tailed tests evaluated at the 0.05 significance level.

### 2.7. Ethical Approval

All subjects gave their informed consent for inclusion before they participated in the study. The study was conducted in accordance with the Declaration of Helsinki, and the protocol was approved by the Ethics Committee of the West China Hospital, Sichuan University (Project identification code: 2012BAI01B02). 

## 3. Results

### 3.1. Participant Characteristics

A total of 19,711 students completed the CBCL questionnaire; 98.9%, 42.43%, and 57.7% were of Han ethnicity and from urban, and rural regions, respectively. There were a higher number of boys (50.42%) relative to girls (49.58%). The population rates of the four cities ranged from 23.98% to 25.93%. In addition, 37.2% of participants were left-behind children, among whom 29.6% and 7.6% were from rural and urban regions, respectively. Approximately 6.8% of children lived alone. Parental educational level was primarily that of junior high school, which accounted for 41.6–43.7% of parents. Furthermore, 3.3% and 1.4% of participants reported their families’ economic status as excellent and very poor, respectively, and 6.9% reported single-parent families. In addition, 1.3% of the children had serious physical illnesses, 40.8% had one or more siblings, and only 0.7% reported family history of mental disease. 

### 3.2. Detection of Behavioral Problems in Children

Behavioral problems were detected in 3769 children, a detection rate of 19.12%. Approximately 61.5% of students’ parents completed the CBCL, of whom 18.5% provided positive ratings. For 25.7% of students, caregivers other than parents completed the CBCL, of whom 23.4% provided positive ratings; 12.8% of students completed the CBCL themselves, and 13.52% provided positive ratings (χ^2^ = 121.21, *p* < 0.05). As shown in Table 1, there was a statistically significant difference in detection rates for mental disorders between boys and girls (21.78% and 16.42%, respectively, χ^2^ = 91.271, *p* < 0.01). Detection rates for all regions ranged from 16.94% to 21.22%, with the highest observed in Bazhong; in addition, rates differed significantly between cities (χ^2^ = 32.74, *p* < 0.05). Furthermore, detection rates were highest in students aged 8–10 (20.16%) and 14–16 (20.60%) years and lowest in those aged 6–7 years (14.23%) and differed significantly according to age group (χ^2^ = 51.98, *p* < 0.05). It is notable that a higher number of left-behind children had been diagnosed with behavioral problems relative to those under parental supervision (χ^2^ = 1419.04, *p* < 0.01). Detection rates did not differ between urban and rural residence (19.42% and 18.71%, respectively, χ^2^ = 1.56, *p* = 0.213). 

**Table 1:** The distribution of gender, age, community, caretaker and cities of positive CBCL (n = 3769).

Variable	Sample	Rate (%)	χ^2^	*p* Value	Cramer’s V
Gender **					
Boys	2164	21.78	91.27	0.003	0.068
Girls	1605	16.42			
Age *					
6–7	356	14.23	51.98	0.026	0.051
8–10	898	20.16			
11–13	1123	18.73			
14–16	1392	20.60			
Community					
Rural	2204	19.42	1.56	0.213	0.009
Urban	1565	18.71			
Caregiver **					
Parents	1362	11.00	1419.04	0.001	0.268
Left-behind	2407	32.83			
Cities *					
Chengdu	801	16.95	32.47	0.014	0.041
Neijiang	924	18.36			
Dazhou	959	19.82			
Bazhong	1085	21.22			

* *p* < 0.05, ** *p* < 0.01.

### 3.3. Overall Point Prevalence Distribution of Mental Disorders

Twenty-three types of mental disorder were diagnosed in 3003 children. Point prevalence was 15.24%, (95% CI: 15.49–16.97; Table 1). Positive CBCL rates were observed for 2929 students diagnosed with mental disorders via the MINI-KID (82.83%); negative CBCL rates were observed for 74 students, with a false negative rate of 7.4%. In addition, 62.7% students and their guardians completed both versions of the MINI-KID; 73.4% of these students were diagnosed with mental disorders. Furthermore, 22.2% of caregivers completed the parent’s version of the MINI-KID alone; 61.3% of the cases were diagnosed with mental disorders. The remainder of the students completed the children’s MINI-KID alone, 56.4% of whom were diagnosed with mental disorders (χ^2^ = 105.14, *p* < 0.05). There were 3291 students diagnosed with mental disorders via the MINI-KID; 3003 of these diagnoses were confirmed by psychiatrists, with a kappa value of 0.91. Of the individual mental disorders, ADHD was the most prevalent (up to 5.37%, 95% CI: 5.06–5.69), followed by ODD (3.01%, 95% CI: 2.78–3.25), TD (2.03%, 95% CI: 1.84–2.23), and major depressive disorder (MDD; 1.90%, 95% CI: 1.71–2.09). Within each disorder category, ADHD and DBD were most prevalent (8.42%, 95% CI 8.03–8.80), followed by anxiety disorders (AD; 3.94%, 95% CI: 3.67–4.21) and affective disorders (2.99%, 95% CI 1.35–3.22; Table 2).

**Table 2:** Point prevalence of DSM-IV disorders in 6–16 years old students (n = 19,711).

DSM-IV Disorders	n	%	95% CI
Any disorders	3003	15.24	14.74–15.74
Affective disorders	588	2.99	1.35–3.22
Major depressive disorder	375	1.90	1.71–2.09
Dysthymia	54	0.27	0.20–0.35
Mania or hypomania	159	0.81	0.68–0.93
Anxiety disorders	777	3.94	3.67–4.21
Panic disorder	29	0.15	0.09–0.19
Agoraphobia without panic	15	0.08	0.04–0.11
Separation anxiety disorder	95	0.48	0.39–0.58
Social phobia	138	0.70	0.58–0.82
Specific phobia	23	0.12	0.07–0.16
Obsessive compulsive disorder	215	1.09	0.96–1.24
Posttraumatic stress disorder	36	0.18	0.12–0.24
Generalized anxiety	226	1.15	0.98–1.30
Substance use disorders	199	1.01	0.87–1.14
Alcohol dependence	89	0.45	0.36–0.55
Alcohol abuse	12	0.06	0.03–0.10
Substance dependence	79	0.40	0.32–0.48
Substance abuse	19	0.10	0.06–0.14
Tic disorder	401	2.03	1.84–2.23
Tourette disorder	52	0.26	0.19–0.34
Chronic motor tic disorder	75	0.38	0.29–0.47
Chronic vocal tic disorder	74	0.38	0.29–0.47
Transient tic disorder	200	1.01	0.88–1.54
Disruptive disorders	1659	8.42	8.03–8.80
ADHD disorder	1059	5.37	5.06–5.69
ADHD Combined	282	1.43	1.24–1.60
ADHD Inattentive	630	3.20	2.95–3.44
ADHD Hyperactive-impulsive	147	0.75	0.63–0.87
Conduct disorder	299	1.52	1.35–1.69
Oppositional defiant disorder	594	3.01	2.78–3.25
Other mental disorders	240	1.22	1.06–1.37
Psychotic disorders	11	0.06	0.02–0.09
Anorexia nervosa	23	0.12	0.07–0.16
Bulimia nervosa	158	0.80	0.68–0.93
Adjustment disorder	42	0.21	0.15–0.28
Pervasive developmental disorder	6	0.03	0.00–0.05
Comorbidity			
Exactly 1 disorder	1908	9.68	9.27–10.09
Exactly 2 disorders	1035	5.25	4.94–5.56
3 or more disorders	60	0.30	0.23–0.38

### 3.4. Gender, Age and Urban-Rural Distribution of Disorders

As shown in Table 3, the overall point prevalence rate for boys (17.33%, 95% CI: 16.59–18.08) was higher than that of girls (13.11%, 95% CI: 12.43–13.77). Prevalence rates for mood and other mental disorders were higher for girls (3.43%, 95% CI: 3.07–3.79 and 2.19%, 95% CI: 1.90–2.48, respectively) relative to boys. However, prevalence rates for other disorder categories were higher for boys relative to girls. Risk of substance use disorder was also higher in boys relative to girls (OR = 7.53, 95% CI: 4.88–11.61, *p* < 0.01).

**Table 3:** Comparison of the prevalence and odds ratio of main groups of mental disorders between male and female.

Genger	Male (n = 9938)	Female (n = 9773)	Odds Ratio (95% CI)	*p* Value
Affective disorders	2.55 (2.24–2.86)	3.43 (3.07–3.79)	0.74 (0.63–0.87)	0.001 **
Anxiety disorders	3.98 (3.60–4.37)	3.90 (3.51–4.28)	1.02 (0.89–1.17)	0.756
Disruptive disorders	11.55 (10.92–12.18)	5.23 (4.79–5.67)	2.21 (2.00–2.44)	0.001 **
Substance disorders	1.77 (1.51–2.03)	0.24 (0.14–0.33)	7.53 (4.88–11.61)	0.001 **
Tic disorders	2.40 (2.10–2.71)	1.66 (1.40–1.91)	1.45 (1.19–1.77)	0.001 **
Other disorders	0.26 (0.16–0.36)	2.19 (1.90–2.48)	0.12 (0.08–0.18)	0.001 **
Any disorders	17.34 (16.59–18.08)	13.10 (12.43–13.77)	1.32 (1.24–1.42)	0.001 **

* *p* < 0.05, ** *p* < 0.01.

The prevalence rate for mental disorders was higher for the 8–10 age group relative to other age groups, with the highest and lowest prevalence observed for children aged nine years (17.11%, 95% CI: 15.18–19.04) and six years (13.52%, 95% CI: 11.51–15.53). The prevalence rates for affective and substance use disorders were highest in participants aged 16 years (8.23%, 95% CI: 7.03–9.42 and 4.00%, 95% CI: 3.14–4.84 respectively). The prevalence rates for anxiety disorders (AD) and tic disorders (TD) were highest in participants aged 10 (6.21%, 95% CI: 5.07–7.34) and nine years (3.56%, 95% CI: 2.61–4.51), respectively. The prevalence of ADHD and DBD were highest in participants aged eight years (12.60%, 95% CI: 10.77–14.45; Table 4).

**Table 4:** Distribution of age for main groups of mental disorders.

Age	Anxiety Disorders	Affective Disorders	Substance Disorders	Tic Disorders	Disruptive Disorders	Other Disorders	Any Disorders
6years	1.79 (1.01–2.57)	1.07 (0.47–1.68)	0 (0–0)	2.06 (1.23–2.90)	9.94 (8.18–11.69)	0.27 (0.04–0.57)	13.52 (11.51–15.53)
7years	1.37 (0.76–1.99)	1.95 (1.22–2.68)	0 (0–0)	3.10 (2.19–4.02)	9.82 (8.25–11.39)	0.36 (0.04–0.68)	14.44 (12.59–16.29)
8years	4.47 (3.32–5.61)	1.27 (0.66–1.90)	0 (0–0)	3.03 (2.08–3.98)	12.60 (10.77–14.45)	0.32 (0.06–0.63)	16.36 (14.31–18.41)
9years	5.00 (3.88–6.11)	1.51 (0.88–2.13)	0.14 (0.05–0.33)	3.56 (2.61–4.51)	11.29 (9.67–12.92)	0.41 (0.08–0.74)	17.11 (15.18–19.04)
10years	6.21 (5.07–7.34)	1.55 (0.97–2.13)	0.63 (0.26–1.00)	3.74 (2.84–4.63)	10.86 (9.40–12.33)	0.17 (0.02–0.37)	16.55 (14.80–18.30)
11years	5.41 (4.41–6.41)	1.12 (0.66–1.59)	0.51 (0.19–0.83)	3.01 (2.25–3.71)	9.95 (8.63–11.28)	0.31 (0.06–0.55)	15.01 (13.42–16.60)
12years	3.52 (2.68–4.37)	1.90 (1.27–2.52)	0.65 (0.28–1.02)	3.25 (2.44–4.06)	10.51 (9.11–11.92)	0.81 (0.40–1.22)	15.99 (14.32–17.67)
13years	3.74 (2.94–4.53)	2.83 (2.13–3.52)	0.59 (0.27–0.91)	1.87 (1.30–2.49)	9.17 (7.96–10.37)	2.23 (1.62–2.85)	15.60 (14.08–17.11)
14years	3.42 (2.69–4.15)	2.95 (2.27–3.64)	1.05 (0.64–1.47)	0.42 (0.16–0.68)	6.84 (5.82–7.85)	2.87 (2.20–3.54)	14.77 (13.33–16.20)
15years	3.56 (2.81–4.31)	5.43 (4.51–6.34)	1.91 (1.36–2.46)	0.17 (0.03–0.34)	3.60 (2.85–4.36)	1.65 (1.14–2.17)	12.68 (11.34–14.02)
16years	4.08 (3.23–4.95)	8.23 (7.03–9.42)	4.00 (3.14–4.84)	0.30 (0.05–0.53)	3.10 (2.35–3.86)	2.07 (1.45–2.69)	16.21 (14.60–17.81)
sum	3.94 (3.67–4.21)	2.98 (2.75–3.22)	1.01 (0.87–1.15)	2.03 (1.84–2.23)	8.42 (8.03–8.80)	1.21 (1.06–1.37)	15.24 (14.74–15.74)
*χ^2^*	82.55	326.55	262.55	182.81	235.66	153.54	25.69
*p*	0.001 **	0.001 **	0.001 **	0.001 **	0.001 **	0.001 **	0.004 **

* *p* < 0.05, ** *p* < 0.01.

The overall point prevalence for rural residence (15.83%, 95% CI: 15.16–16.51) was significantly higher than that for urban residence (14.42%, 95% CI: 13.67–15.17; χ^2^ = 7.46, *p* = 0.006). The prevalence rates for ADHD and DBD (9.49%, 95% CI: 8.87–10.12) and TD (2.45%, 95% CI: 2.12–2.78) were higher in participants from urban regions relative to those from rural regions (7.62%, 95% CI: 7.13–8.11; 1.73%, 95% CI: 1.49–1.97, respectively). There was a higher risk of substance use disorders in participants from rural regions relative to those from urban regions (OR = 3.34, 95% CI: 2.33–4.78, *p* < 0.01; Table 5).

**Table 5:** Comparison of the prevalence and odds ratio of main groups of mental disorders between in rural *vs.* in urban areas.

Community	Rural (n = 11,348)	Urban (n = 8363)	Odds Ratio (95% CI)	*p* Value
Affective disorders	3.89 (3.53–4.24)	1.76 (1.48–2.04)	2.21 (1.84–2.66)	0.001 **
Anxiety disorders	3.91 (3.56–4.27)	3.98 (3.56–4.40)	0.98 (0.86–1.13)	0.805
Disruptive disorders	7.62 (7.13–8.11)	9.49 (8.87–10.12)	0.80 (0.73–0.88)	0.001 **
Substance disorders	1.43 (1.22–1.66)	0.43 (0.29–0.57)	3.34 (2.33–4.78)	0.001 **
Tic disorders	1.73 (1.49–1.97)	2.45 (2.12–2.78)	0.71 (0.58–0.86)	0.001 **
Other mental disorders	1.66 (1.42–1.89)	0.62 (0.45–0.79)	2.66 (1.96–3.62)	0.001 **
Any disorders	15.84 (15.16–16.51)	14.42 (13.67–15.17)	1.10 (1.03–1.18)	0.006 **

* *p* < 0.05, ** *p* < 0.01.

### 3.5. Prevalence of Mental Disorders in Left-Behind Children

A chi-square test was performed to examine mental disorders in left-behind children according to city. Pairwise comparison revealed that the prevalence of left-behind children with mental disorders in Bazhong city differed significantly from those of the other cities (χ^2^ = 273.04, *p* < 0.01). As shown in Table 6, prevalences of different disorder categories were higher in left-behind children relative to those under parental supervision, suggesting a significant correlation between left-behind-status and prevalence of mental disease; the OR value was greater than 1, with *p* < 0.01.

**Table 6:** Comparison of the prevalence and OR of main groups of mental disorders between left-behind and parents in Sichuan province.

Caregiver	Left-Behind (n = 7331)	Parents (n = 12,380)	Odds Ratio (95% CI)	*p* Value
Affective disorders	4.50 (4.03–4.98)	2.08 (1.83–2.34)	2.22 (1.88–2.61)	0.001 **
Anxiety disorders	6.55 (5.98–7.11)	2.40 (2.13–2.67)	2.85 (2.46–3.30)	0.001 **
Disruptive disorders	15.32 (14.49–16.14)	4.33 (3.97–4.69)	3.99 (3.59–4.45)	0.001 **
Substance disorders	1.64 (1.35–1.92)	0.64 (0.50–0.78)	2.59 (1.95–3.45)	0.001 **
Tic disorders	4.17 (3.72–4.63)	0.77 (0.61–0.92)	5.63 (4.47–7.10)	0.001 **
Other disorders	1.69 (1.40–1.99)	0.94 (0.77–1.11)	1.82 (1.41–2.35)	0.001 **
Any disorders	26.42 (25.42–27.43)	8.61 (8.11–9.10)	3.81 (3.51–4.14)	0.003 **

* *p* < 0.05, ** *p* < 0.01.

### 3.6. Distribution of Mental Disorders According to City

Table 7 shows the results of the chi-square tests for each category of disorder according to city. There were significant differences in all disorder categories between the four cities, with the exception of AD and other mental disorders. Further, the prevalence rate for AD was slightly higher in Neijiang (4.19%, 95% CI: 3.64–4.75) relative to Bazhong (4.15%, 95% CI: 3.60–4.69), while the prevalence rates for all other categories were higher in Bazhong city relative to the other cities.

**Table 7:** Distribution of the prevalence of main groups of mental disorders in four cities of Sichuan province.

Cities	Chengdu (n = 4727)	Neijiang (n = 5033)	Dazhou (n = 4838)	Bazhong (n = 5113)	*p* Value
Affective disorders	2.98 (2.50–3.47)	2.44 (2.02–2.87)	2.65 (2.19–3.10)	3.83 (3.31–4.36)	0.001 **
Anxiety disorders	3.45 (2.93–3.97)	4.19 (3.64–4.75)	3.95 (3.40–4.50)	4.15 (3.60–4.69)	0.218
Disruptive disorders	5.80 (5.13–6.46)	8.54 (7.77–9.32)	9.32 (8.50–10.14)	9.86 (9.04–1067)	0.001 **
Substance disorders	1.10 (0.80–1.40)	0.70 (0.47–0.93)	0.50 (0.30–0.69)	1.72 (1.36–2.10)	0.001 **
Tic disorders	1.95 (1.55–2.34)	1.47 (1.14–1.80)	2.34 (1.91–2.76)	2.39 (1.97–2.80)	0.004 **
Other disorders	1.14 (0.84–1.45)	1.21 (0.91–1.51)	1.16 (0.86–1.46)	1.35 (1.03–1.67)	0.775
Any disorders	12.61 (11.66–13.56)	14.54 (13.57–15.52)	15.50 (14.48–16.52)	18.09 (17.04–19.15)	0.001 **

* *p* < 0.05, ** *p* < 0.01.

### 3.7. Comorbidity

The number of participants with two comorbid disorders was 1,095, which accounted for 36.46% of students with mental disorders. Of these, 60 had been diagnosed with three comorbid disorders, accounting for only 1.93% of students with mental disorders. The most common comorbid disorder was ADHD, with 42.14%, 38.33%, and 25.25% of students with ADHD diagnosed with comorbid conduct disorder (CD), generalized anxiety disorder, and oppositional defiant disorder (ODD), respectively. The prevalence of comorbid major depressive disorder (MDD) and tic disorder (TD) was 14.67%.

## 4. Discussion

### 4.1. Point Prevalence

In this study, 3769 children with behavioral problems were identified in valid data from 19,711 primary and middle school students (detection rate: 19.13%). The CBCL was completed by parents or other caregivers, and if they were unavailable, students completed the CBCL for themselves. The rate of positive CBCL ratings from parents was lower than that of other caregivers but higher than of the students; it is possible that students cared for by other caregivers received less concern and nurturing from their parents, resulting in mental health problems. However, the differences of positive CBCL ratings between parents and students probably caused by two broadband scales (Internalizing and Externalizing); self-report is higher in internalizing problems, whereas parents report is higher in externalizing problems, which is consistent with many studies. We also noticed that the MINI-KID combined both versions for diagnosis had higher sensitivity than using either version alone. There were a higher number of boys than girls. In addition, the detection rate for mental disorders is consistent with data from domestic and foreign studies [9,27]. Twenty-three types of mental disorder were diagnosed in 3003 participants, giving a total point prevalence of 15.24%. Multi-disease epidemiological studies adopting a two-phase methodology conducted in other countries in the last 10 years have reported overall prevalence rates of 7.0%–19.8% for DSM-IV mental disorders in children, which are consistent with the results of this study [33,34]. The results of this study are consistent with those of a study conducted in Hunan in 2005 examining mental disorders in primary and middle school students, but significantly higher than that of a similar study conducted in Liaoning in 2007 [27,28]. This inconsistency may be due to differences in diagnostic tools and the regions included.

ADHD was the most prevalent disorder, accounting for 5.37% of all disorders, which is slightly lower than the prevalence of 6% in children between 7 and 11 years old reported in a recent meta-analysis [35]; significantly lower than the rate of 13.6% reported in a domestic study conducted by Lu *et al.* (13.6%) involving participants aged 4–16 years, and consistent with the rate of 5.4% reported in a study conducted by Zhang *et al.* involving six cities [36,37]. The variance in socio-economic status, cultural practices, and parental education methods between countries could influence children’s personalities and psychology, resulting in variance in ADHD prevalence rates between countries. In addition, the most common disorders within the spectrum of AD were obsessive-compulsive disorder and generalized anxiety disorder, both with prevalence rates of more than 1% [38,39]; however, the most common disorders in studies conducted in other countries were separation anxiety and overanxious disorder. This difference may be related to the fact that Chinese students suffer greater academic stress, due to greater pressure from parents and teachers. 

### 4.2. Sex and Age

Sex comparison revealed that the overall prevalence of mental disorders was higher in boys relative to girls, which was consistent with similar surveys [7,8,9,10,11]. This was primarily because the prevalence of ADHD and DBD was significantly higher in boys relative to girls. Boys also showed significantly higher substance dependence relative to girls, with drinking and smoking the major types of substance dependence. This may be due to environments involving high rates of adult drinking and smoking, which are related to adolescent depression and anxiety. The most common disorder in girls was anxiety depressive disorder, and girls aged 15–16 years experienced higher rates of depression and eating disorders, which is consistent with results from other domestic and foreign studies [27,34]. Regarding age distribution, although rates of all types of mental disorder were lowest in children aged six years, overall prevalence rates exceed 10%, particularly for AD and MDD, which were both beyond 1%. This may have been due to children attending new schools and meeting new friends, which may have caused maladjustment; in addition, children may have misunderstood the questions asked by psychiatrists due to their young age, and parents may have paid too much attention to them, leading to false positive results. The prevalence of mental disorders in 16-year-old participants (16.21%) was significantly higher than that of those aged 15 years (12.68%), which is inconsistent with previously reported results [10,27]; this was mainly due to the significantly higher proportion of 16-year-old adolescents suffering from mood disorders and substance dependence relative to 15-year-olds. This could have been related to the transition to high school in the older group, as they face the following: (1) a new environment (e.g., leaving home and contact with new students and teachers), leading to maladaptive or emotional disorders; (2) greater study pressures (e.g., more difficult courses, college entrance examination); and (3) further personality development, leading to romantic interests and imitation of drinking and smoking behaviors. Foreign studies report average mental disorder prevalence rates of 13.2% and 16.5% in children aged 6–12 years and 13 years or older, respectively [5]. This survey revealed a similar prevalence rate for children aged 12–16 years, but a higher overall rate for those aged 6–11 years. This discrepancy may be because ADHD, DBD, and TD mainly occur within this age range in Chinese children. Puberty may have been the reason for the higher prevalence in students aged 12–16 years, as they were undergoing transition from childhood to adulthood, and adolescents not only experience physical and mental changes, but also face other adverse factors that lead to mental health problems.

### 4.3. Regional Differences and Issues for Left-Behind Children

The prevalence rate for mental disorders was higher in children from rural regions relative to urban regions, which is consistent with findings from several domestic studies and may have occurred because the economy, education, and healthcare in rural districts lags behind that of cities in China [27]. Prevalence rates also differed significantly between the four cities, which had different economic levels. The prevalence rate for mental disorders was significantly higher in economically undeveloped regions relative to developed regions. With China’s reform and progression, millions of people have relocated from rural to urban regions to work. Following this rapid increase in migrant workers, the number of left-behind children has also risen. Left-behind children were defined as “children younger than 18 years of age who have been left behind at their original residence for at least 6 months while one or both parents migrate to other areas to work.” [40] Approximately 58 million children younger than 18 years of age were estimated to have been left in rural hometowns by their parents in 2008. China Network News has reported that left-behind children in six provinces account for 52% of all left-behind children, particularly in western areas including Sichuan [41].

The prevalence of any mental disorder was significantly higher in left-behind children relative to those under parental supervision in each group, and left-behind children in rural regions demonstrated more severe mental health issues. 

The issue of children being left behind is a unique and serious social problem in China, particularly in undeveloped western areas [42,43,44]. This study included participants from the most populated province in western China, which has relatively low levels of economic and cultural development. Of the four cities selected, Chengdu, as the capital city, is developed economically; Neijiang and Dazhou are relatively less developed, and Bazhong city is the least developed area and has the lowest GDP per capita in the province, with poor education and healthcare systems. Most parents work outside the city, and many children are left at home. Lack of parental care and an undesirable home environment can lead to mental and behavioral abnormalities in children.

### 4.4. Comorbidity

In this study, 36.46% of children and adolescents displayed disorder comorbidity; in particular, the rate of comorbidity was highest for ADHD, with approximately 10%, 5.25%, and 0.3% of children with ADHD diagnosed with 1, 2, and 3 comorbid mental disorders, respectively. Merkangas found that approximately 25%, 11%, and 3% of adolescents were diagnosed with at least 1, 2, and 3 mental disorders, respectively [38]. In a longitudinal study, Castello found that 25.5% of children with ADHD were diagnosed with two or more comorbid mental disorders [12]. As children with ADHD possess poor self-control and high impulsivity, they enter into conflict with peers, teachers, and parents easily and are prone to depression, obsessive-compulsiveness, panic attacks, negativism, violation of discipline, and other emotional and behavioral problems [45]. Other commonly observed comorbidities in the present study included ODD, CD, TD, and MDD. This result is consistent with findings of a domestic study conducted by Sun *et al.* [46] and probably occurred due to a common pathogenesis between these disorders. The fact that some of these disorders share certain symptoms cannot be excluded, which leads to diagnosis extension. 

### 4.5. Limitations

This study was subject to a number of limitations. First, we did not compare standard schools with key schools, as the inconsistencies between them are difficult to identify. Second, CBCL is an old questionnaire with undesirable sensitivity, which can cause higher rates of missed diagnosis. The MINI-KID is simple to understand and easy to administer but is not commonly used in domestic research, preventing comparison with similar studies. In addition, it has relatively low validity for the diagnosis of affective disorders and emotional problems in children, which may have affected diagnostic accuracy in certain disorders [31]. Third, the sample should have included participants from the western region of Sichuan; however, participants from this area were in the minority, mainly due to a language barrier. Fourth, some students aged 6–16 years may not have been included in the study, as they did not attend grades 6–10.

## 5. Conclusions

Stratified cluster sampling was performed in four Sichuan regions, which were representative of the different levels of economic development in the province, and a two-phase epidemiological survey method was adopted to investigate the prevalence of mental disorders. The first phase involved screening, and the second phase involved formalized psychiatric interviews and clinical diagnoses based on the DSM-IV diagnostic criteria. Quality control was performed during the study. Therefore, the study reflected objective point prevalence of mental disorders in primary and middle school students in Sichuan.

The distribution pattern of this survey suggests important mental health issues and a high level of mental disorder comorbidity in children in this age group in Sichuan. This is a serious problem that may harm the physical and mental health of children and adolescents. In particular, the mental health of left-behind children and adolescents in economically undeveloped regions should not be overlooked. 
